# *Galla chinensis* alleviated liver damage induced by acetaminophen by regulating intestinal microbiota

**DOI:** 10.3389/fmicb.2025.1589946

**Published:** 2025-07-01

**Authors:** Xiaocong Li, Jiadong Chen, Chang Xu, Yong Li, Kun Li

**Affiliations:** ^1^Hubei Three Gorges Polytechnic, Yichang, China; ^2^College of Veterinary Medicine, Nanjing Agricultural University, Nanjing, China

**Keywords:** *Rhus chinensis* Mill., microbiota, inflammation, metabolomics, mice

## Abstract

**Introduction:**

Drug-induced liver injury is a common condition of therapeutics and requires solutions other than drugs. Dietary supplements and herbal products have beneficial potential against liver failure and are often associated with changes in intestinal flora. This study was designed to investigate *Galla chinensis* (GC) potential in alleviating drug-induced liver injury.

**Methods:**

The treatment group and model group were administered 0.5 mL of GC oil daily for 14 days, followed by induction of acute drug-induced liver injury using Acetaminophen (APAP) on the 15th day via intraperitoneal injection. Serum biochemical analysis and intestinal bacteria 16S rRNA sequencing were conducted to explore the anti-drug liver injury activity of GC.

**Results:**

The results showed that GC treatment alleviated the increased liver organ index caused by APAP. Furthermore, serum biochemical analysis revealed that GC therapy reduced alanine transaminase (ALT) and aspartate transaminase (AST) levels, indicating a protective effect against APAP-induced liver injury. Additionally, GC demonstrated the ability to regulate the diversity and composition of intestinal microbes and help increase the prevalence of beneficial microbes such as *Faecalibaculum* and *Odoribacter* while reducing the prevalence of pathogenic bacteria, including *Corynebacterium, Rikenella*, *Bacillus, Phocaeicola*, and *Alloprevotella*.

**Discussion:**

Our findings indicate that GC plays a significant role in mitigating drug-induced liver injury by improving gut microbiome composition, reducing plasma ALT and AST levels, and inhibiting oxidative stress and inflammatory responses. The study further demands investigations to make pharmaceutical products to be used as nutraceuticals against drug-induced toxicities.

## 1 Introduction

The liver, among other body organs, holds a superior role in terms of physiological functions in synthesis, metabolism, detoxification, and immunity (Devarbhavi et al., 2023). Hepatic dysfunction is responsible for approximately two million deaths worldwide per annum, with acute liver injury (ALI) constituting one of the major underlying causes. Acute liver injury refers to a class of clinical conditions that cause rapid liver function disorders within 2 weeks of exposure to various risk factors in individuals without liver disease or with stable liver disease (Yang et al., 2023). Drug-induced liver injury accounts for about 50% of acute liver injury cases in the United States (Sherlock et al., 2022). This harm can be dose-dependent and predictable. Specific drug-induced liver damage is relatively rare, even for patients exposed to potentially hepatotoxic drugs. Hepatic toxicity caused by Acetaminophen (APAP) is the most common manifestation of acute liver injury in the United States.

Acetaminophen is well tolerated at reasonable therapeutic doses with few adverse effects (Zafeiri et al., 2021), making it the first choice in treating the COVID-19 pandemic. However, an overdose of APAP can cause severe acute liver injury. At usual doses, acetaminophen is absorbed and metabolized into water-soluble substances mainly in the liver through glucosidation and sulfation (*O*-glucuronic acid), 55% of the dose, and O-sulfate, 30% of the dose, and is excreted in the urine (McGill and Hinson, 2020). A smaller portion (10–15%) is hydroxylated by cytochrome P450 (isoenzymes CYP1A2, CYP2E, CYP3A4) to form the powerful alkylated para-acetylbenzoquinone imide (NAPQI), which binds to glucuronide and is excreted through bile (Fisher and Curry, 2019). This means that in normal doses, most species, including humans, form only a tiny amount of NAPQI, which is then combined with GSH to form the corresponding GSH adduct (4% of the dose). In such cases, there are no significant adverse effects after taking acetaminophen (Athersuch et al., 2018). However, after an overdose, or when a specific P450 isoenzyme is increased, the liver GSH is consumed by a large amount of NAPQI and can no longer cope with the remaining large amount of NAPQI by compensating. Therefore, mitochondrial GSH depletion is often associated with acetaminophen hepatotoxicity. Therefore, mitochondrial oxidative stress is frequently related to the hepatotoxicity of APAP (Sun Y.-K. et al., 2022). In this context, N-acetyl cysteine acts as an active oxygen scavenger and is often used to treat early APAP-induced ALI, but its use has many limits.

In recent years, as a new research hotspot, Chinese herbal medicine has been widely applied (Lee et al., 2022). *Galla chinensis* (GC) is a gall formed by a hornaphid or egg aphid in the host trees of *Rhus chinensis*, *Populus cyanoplecta*, or *Red populus* (Sun et al., 2023). It is widely distributed in Yunguichuan and Shaanxi Province and is a vital resource of Chinese herbal medicine. Additionally, RCM exerts a broad range of biological properties, including astringent, antibacterial, and antitumor effects (Li et al., 2022). Previous reports revealed that RCM relieves liver injury by regulating glucose and lipid metabolism disorders and colitis (Wu et al., 2021; Zhang et al., 2022). However, studies on RCM against APAP-induced acute liver injury with a perspective on gut microbiota are inadequate.

The gut microbiota plays an important role in maintaining the health of the host, and its balance can be affected by various external factors, including drugs, the environment, and a high-fat diet (Ma and Lee, 2025). The imbalance of these microbes (dysbiosis) leads to an increase in the permeability of the intestinal barrier functions, which travel to the liver due to the close anatomical relationship between the liver and the gut (Dmytriv et al., 2024; Pabst et al., 2023). The liver produces bile acids, which are essential in microbial homeostasis and lipid metabolism. Any disruption in the gut barrier can lead to bacterial translocation, contributing to the worsening of a variety of hepatic disorders such as nonalcoholic fatty liver disease, obesity, and nonalcoholic steatohepatitis (Wang et al., 2025), which can be addressed by use of traditional Chinese Medicine (TCM).

The TCMs are significant attention for their role in modulating the gut-liver axis, which plays an important role in the pathogenesis of various hepatic diseases (Wang Y. et al., 2024). Specifically, a review by Zhang et al. (2020) underscored that Chinese herbal medicines targeting the gut-liver axis can ameliorate hepatic injuries by restoring gut microbiome balance and reducing the levels of inflammatory mediators (Zhang et al., 2020). Additionally, another study by Luo et al. (2024) emphasized the role of TCM in modulating gut microbial composition and their metabolites, which in turn affect hepatic health, suggesting TCM is promising for managing hepatic pathologies through the gut-liver axis (Luo et al., 2024). In this study, we focused on alleviating the effect of RCM on APAP-induced acute liver injury and discussed it from three perspectives: oxidative stress, intestinal flora, and metabolic pathways.

## 2 Materials and methods

### 2.1 Drug extraction

*Galla chinensis* seeds were collected and processed to remove impurities as per standard protocols. The seeds were crushed and mixed with 1 L n-hexane, following which the mixture was kept in an ultrasonic machine at 35°C for 1 h, pumped, filtered, and, pharmaceutical residue and liquid were separated. The process was repeated three times with only a difference in the duration of keeping material in an ultrasonic machine, in that it was 1 h during the initial two repetitions, and 1.5 h during the third repetition. Thereafter, the material was put overnight in a vacuum-drying oven to get the final finished product of oil.

### 2.2 Animal and experimental design

The experimental study involved *n* = 45 ICR mice of 4 weeks of age purchased from Yangzhou University, with cardinal signs of health declared as normal. Following a week of acclimatization, mice (*n* = 15) were randomly allocated into three groups: named as blank (negative) control group (CW), the APAP-induced acute liver injury group (MW), and the GC treatment group (OW). The mice were maintained at 25°C with 50–60% relative humidity and a 12-h light/dark cycle. Standard rodent chow and distilled water were provided *ad libitum* throughout the experiment.

Mice in the OW group were given 0.5 mL of GC oil extract daily, while other mice received the same quantity of olive oil (AGRIC, Spain). The GC dose in the present study was chosen following testing different concentrations and finally agreed on the concentration which was also reported in a previous study (Sun Y. et al., 2022). The selected dose produced promising results with a safe window as an antidote to toxicity. Mice in the MW group and OW group were intraperitoneally injected with 20 mg/mL of acetaminophen at a dose of 300 mg/kg (dissolved in normal saline) while the blank group received 0.5 mL of normal saline. Following 24 h of administration, the mice were sacrificed through cervical dislocation after being injected with ether anesthesia. The liver and intestinal samples were collected for subsequent analysis. Blood samples collected from the posterior orbital venous plexus, before euthanizing, were processed for serum collection and stored at −20°C for subsequent analysis.

### 2.3 Serum biochemical, oxidative stress, and inflammatory factors

Liver function tests (ALT and AST), inflammatory factors (IL-6, IL-10, IL-1β, and TNF-*α*), and markers of oxidative stress (MDA, TAOC, SOD, and GSH-px) were analyzed from serum samples of experimental mice. The test kits for liver function tests were procured from Nanjing Jiancheng Bioengineering Institute, while the kits to test inflammatory factors and oxidative stress were purchased from Jianglai Biotechnology Co., LTD., and Soleibao, respectively.

### 2.4 Total DNA extraction of the intestinal microbiome

Fecal samples from mice were put through DNA extraction using the SolarBio® fecal DNA extraction kit. The quality of extracted DNA was confirmed using Nanodrop (2000), while the quality was determined using a 1.5% agarose nucleic acid gel.

### 2.5 Target fragment PCR amplification

We selected the V3-V4 variable region of the 16S rRNA gene as the target and designed primers according to the conserved region in the sequence to perform PCR amplification. Pfu high-fidelity DNA polymerase was used for PCR amplification, and the number of cycles was strictly controlled to ensure the same amplification conditions for samples. During amplification, a negative control was also run to exclude microbial contamination of the environment and reagents.

### 2.6 Purification of amplified products and bioinformatics analysis

The PCR amplification products were purified and finally recovered through magnetic beads, which were further quantified and detected using Quant-iT PicoGreen dsDNA Assay Kit and BioTek’s FLx800 microplate reader, respectively. QIIME2 was used to filter and denoise the raw sequencing data. Unmatched primer sequences were removed using qiime cutadapt trim-pairs, followed by dada2 de-noising pairs for quality control, de-noising, merging, and checking of chimera to obtain clean data for subsequent analysis. This included *α* diversity analysis (Chao1, Pielou-e, Goods coverage, Simpson, Shannon, Observed-species, and Faith pd) and *β* diversity analysis (PCoA and NMDS). Additionally, R software and LDA Effect Size (LEfSe) were also employed to analyze the gut flora and species composition of different gut microbes (Yu et al., 2022). The abundance of KEGG functional pathways was predicted based on the KEGG database, with differences in metabolic pathways among different groups being analyzed.

### 2.7 Data analysis

The experimental data were analyzed using GraphPad Prism 10.1.2 or R 4.5.0 software and one-way analysis of variance. The results are presented as Mean ± SD. To control for potential false positives arising from multiple hypothesis testing, *p*-values were adjusted using the Bonferroni correction method. The adjustment was performed independently within each biological group (i.e., cytokines and oxidative stress markers) to account for group-specific multiple comparisons. Adjusted p-values are reported, and significance was defined as *p* < 0.05 after correction.

## 3 Results

### 3.1 Impact on liver damage

**Figure 1:**
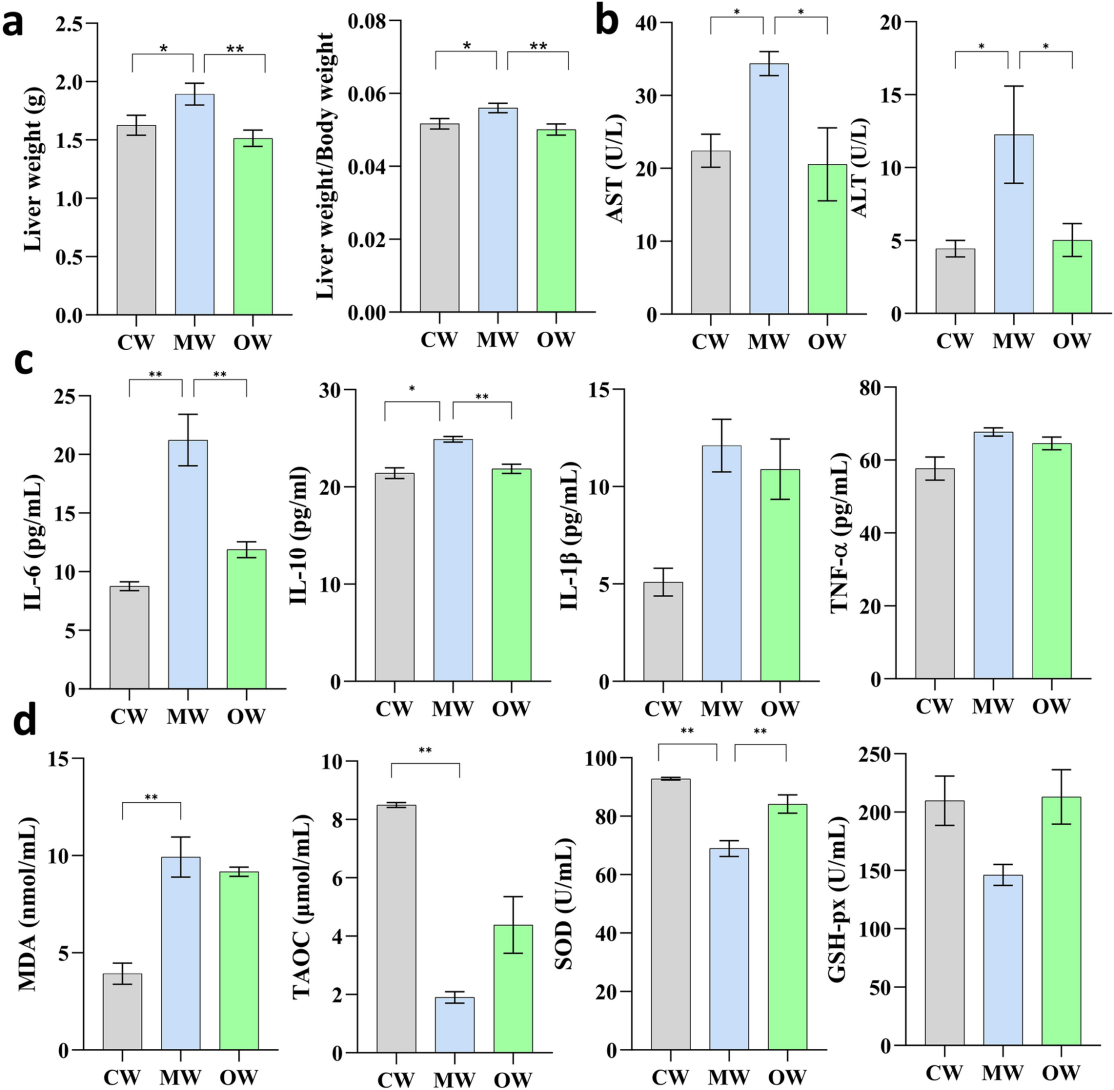
Effects of *Galla chinensis* treatment on liver weight, liver indexes, and serum factor in APAP-induced mice. **(a)** Liver weight and liver weight to body weight ratio, **(b)** liver function tests, cytokines, **(c)** inflammatory mediators, **(d)** antioxidant enzymes. *p < 0.05, **p < 0.01, ***p < 0.001, and ****p < 0.0001; data were presented as the mean ± SD.

The current study found a significant increase in liver weight in the MW group (*p* = 0.045) in the case of APAP-induced, while RCM-treated showed, on the other hand, significantly decreased liver weight (*p* = 0.0032). Interestingly, there was a highly significant increase in liver index in the case of the MW group (*p* = 0.0339) and vice versa in the case of RCM (*p* = 0.0066) (Figures 1a,b).

### 3.2 Role in affecting cytokines, antioxidant indexes, and biochemical markers in mice

There were variable responses found in each treatment when it comes to cytokines, antioxidant indices, and biochemical markers. After Bonferroni correction within each biological group, levels of IL-6 (adjusted *p* = 0.0060) and IL-10 (adjusted *p* = 0.0156) remained significantly increased in the APAP group compared with the control group (CW). In contrast, although IL-1β (adjusted *p* = 0.0704) and TNF-*α* (adjusted *p* = 0.1632) exhibited apparent elevations, these differences were no longer statistically significant after correction. For oxidative stress markers, MDA (adjusted p = 0.0060) and SOD (adjusted *p* = 0.0030) were significantly decreased following APAP induction, and a similar significant reduction was observed for T-AOC (adjusted *p* = 0.0015). Meanwhile, both cytokine and antioxidant enzyme levels tended to normalize in the GC-treated group (Figures 1c,d). Additionally, serum levels of ALT and AST were significantly increased (*p* < 0.05) in the APAP-induced model group and significantly decreased after GC treatment (*p* < 0.05).

### 3.3 Intestinal microbial diversity in mice

**Figure 2:**
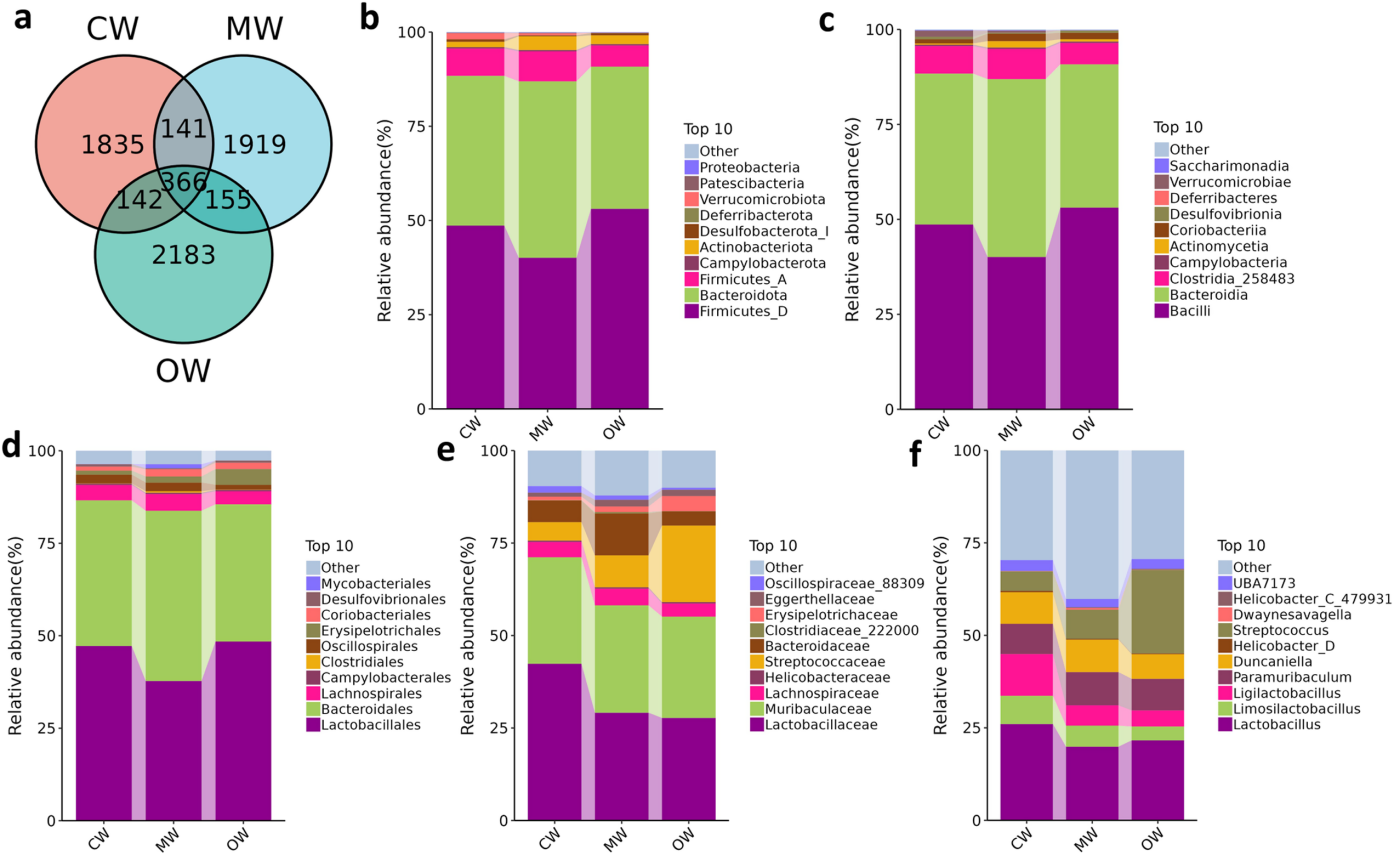
Comparing microbiota composition of mice in different taxa. **(a)** Venn map; **(b)** phylum, **(c)** class, **(d)** order, **(e)** family, **(f)** genus.

**Figure 3:**
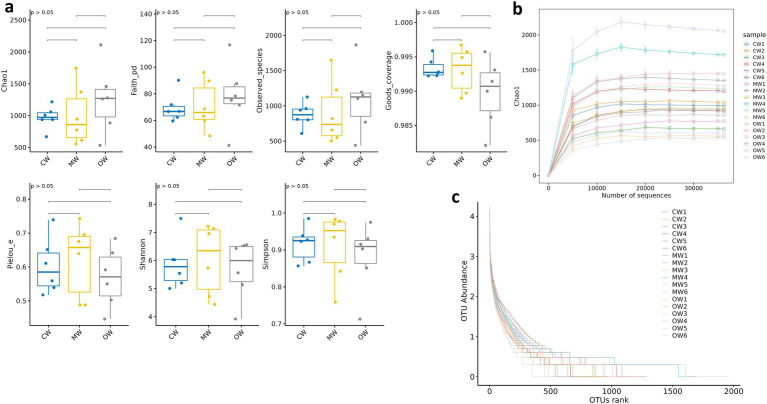
Effects of *Galla chinensis* treatment on alpha diversity analysis of the gut microbiota. **(a)** Alpha diversity index, **(b)** rarefaction curve, **(c)** Rank abundance curve.

A total of 1 phylum and 3 genera exhibited significant variations in abundance among the three groups. While there were no statistically significant differences, 2 phyla and 15 genera showed variations in abundance across the three groups. The current study noted 68,000, 77,000, and 92,000 raw data in the CW, MW, and OW groups, respectively, and after filtering, the data kept enumerated to be 62,000, 71,000, and 81,000 sequences, respectively, for subsequent analysis. These sequences identified 5,937 ASV sequences, of which 366 were shared by three groups (Figure 2a). We performed multiple alpha diversity analyses, including Chao1, Faith_pd, Observed_species, Goods_coverage, Pielou_e, Shannon, and Simpson, and the analysis results show no significant difference (Figure 3a). The rank–abundance curve showed a wider range and smoother shape along the horizontal axis, indicating higher species abundance and evenness (Figure 3b). The rarefaction curve gradually flattened as sequencing depth increased, confirming that the sequencing was sufficient to capture all species in the samples and met the requirements for further analysis (Figure 3c).

At the phylum level, the dominant phyla of group CW, MW, and OW were Firmicutes_A (7.24, 7.94, 5.61%), Bacteroidota (39.72, 46.82, 37.72%) and Firmicutes_D (48.65, 40.10, 53.10%). The current study noted Actinobacteriota occupying a higher proportion in the MW group (3.64%) compared to that of the CW group (1.41%) and OW group (2.30%). It was also noted that Deferribacterota was higher in the GC treatment group. From the class level, the main classes of CW, MW, and OW groups were Bacilli (48.65, 40.10, 53.10%), Bacteroidia (39.73, 46.82, 37.72%) and Clostridia_258483 (7.24, 7.94, 5.61%). At the order level, Lactobacillales took higher percentages in group CW (47.23%) and OW (48.42%), compared to the MW (37.77%) group. The highest percentages in the MW group were Bacteroidales, which also occupied a high proportion in the CW (39.38%) and OW (37.10%) groups. Lachnospirales were found as 4.12, 4.57, and 3.52% in the control, APAP, and GC treatment groups, respectively. Concerning analysis at the family level, Lactobacillaceae (42.37, 29.14, 27.72%) and Muribaculaceae (28.79, 29.02, 27.39%) were found higher in the three groups. In addition, Bacteroidaceae (5.89%) and Lachnospiraceae (4.13%) had a higher percentage in the CW group. In the CW group, Lactobacillus (26.03%) was at the highest percentage at the genus level, followed by Ligilactobacillus (11.27%), Duncaniella (8.54%) and Paramuribaculum (8.18%). At the same time, these three genera also with a relatively high proportion of OW (21.60, 6.67, 8.54%), but the genus with the highest abundance in the OW group was Streptococcus (22.58%) as shown in Figures 2b–f.

### 3.4 Marker bacteria for the remission effect of *Galla chinensis* therapy on ALI

**Figure 4:**
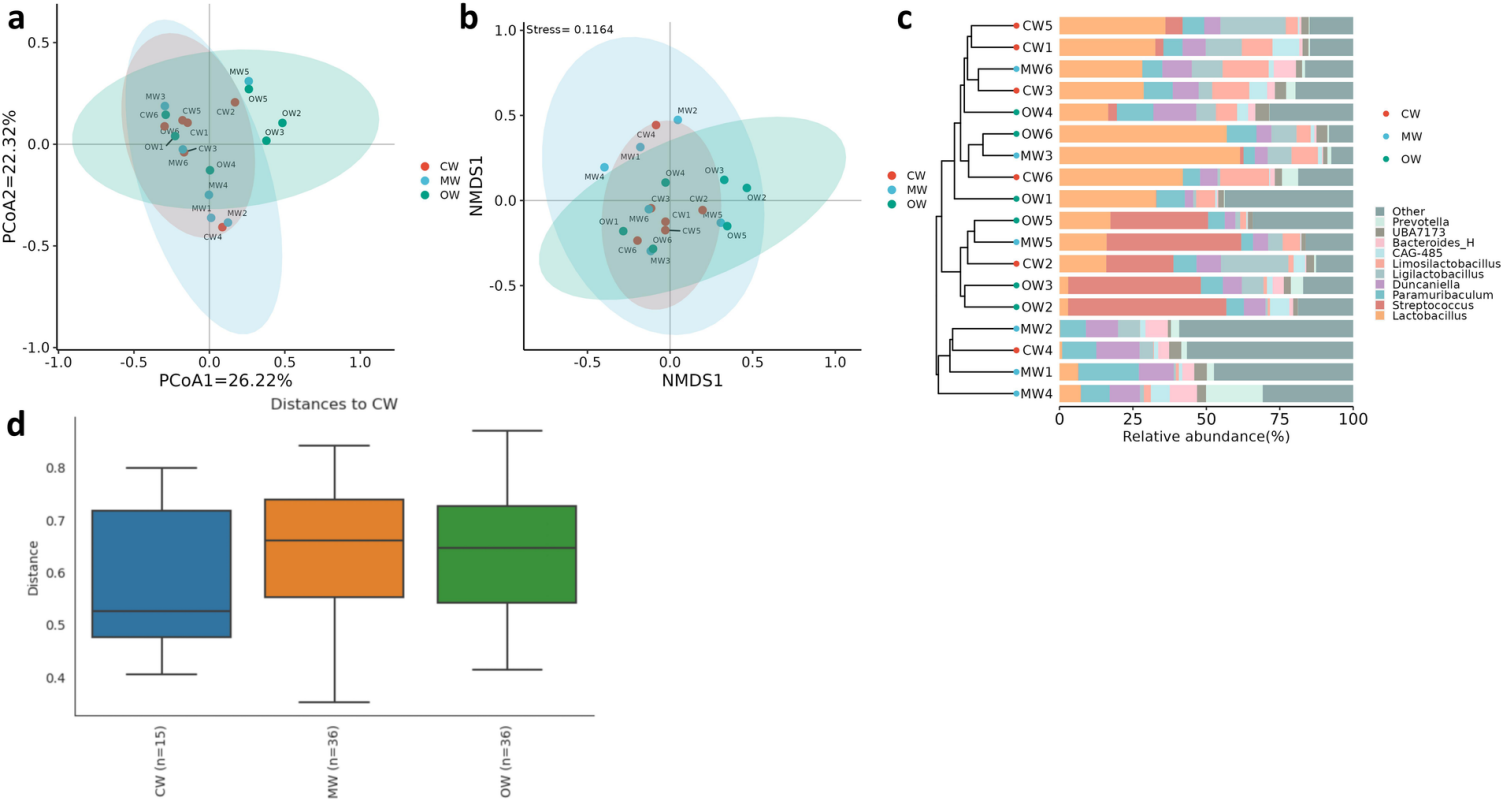
Effects of *Rhus chinensis* Mill. treatment on beta diversity analysis of the gut microbiota. **(a)** PCoA, **(b)** NMDS, **(c)** UPGMA, **(d)** PERMANOVA.

**Figure 5:**
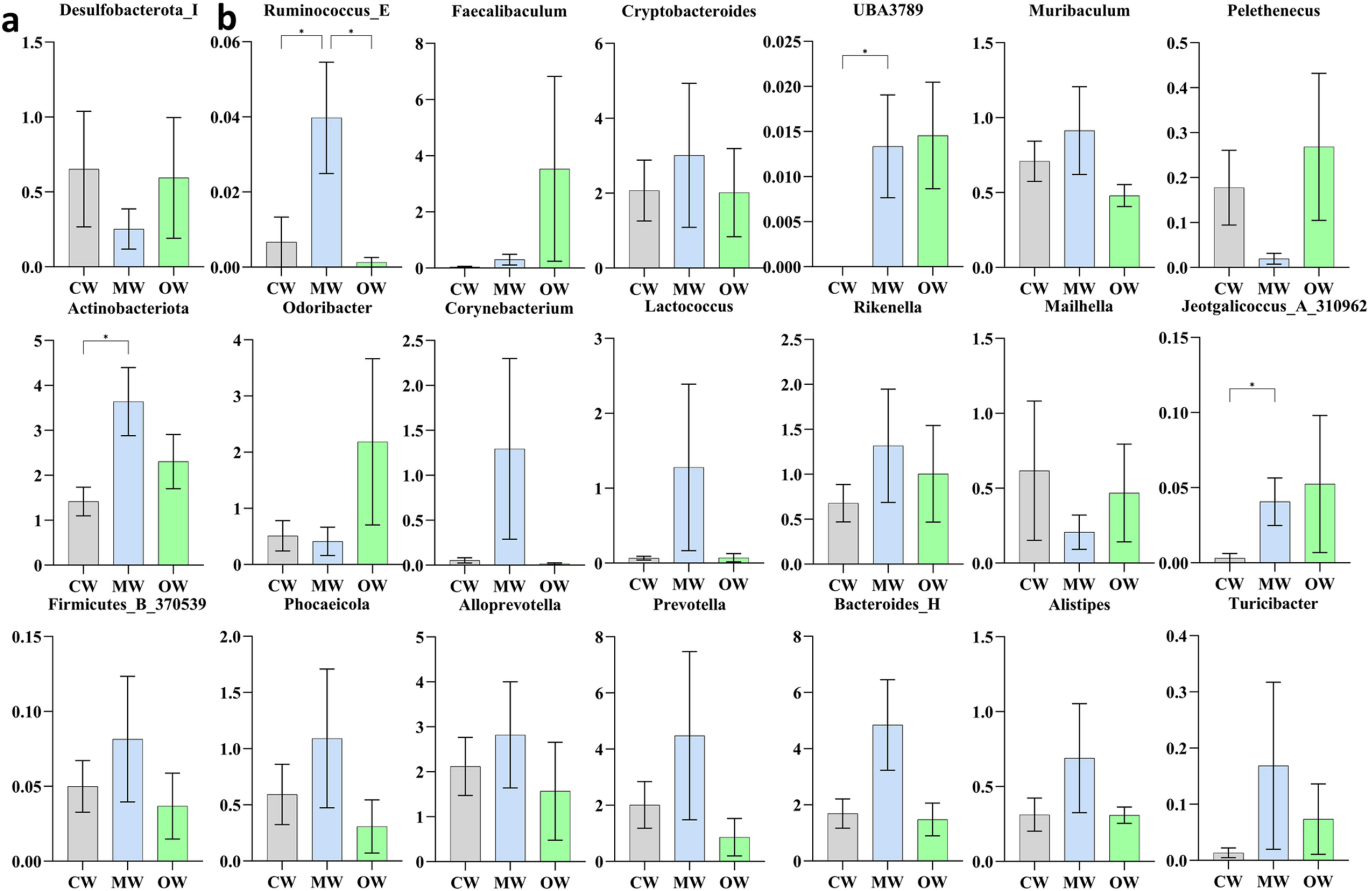
Comparison of significantly altered bacteria among the three groups based on one-way ANOVA analysis. **(a)** Phylum, **(b)** genus. *p < 0.05, **p < 0.01, ***p < 0.001, and ****p < 0.0001; data were presented as the mean ± SD.

**Figure 6:**
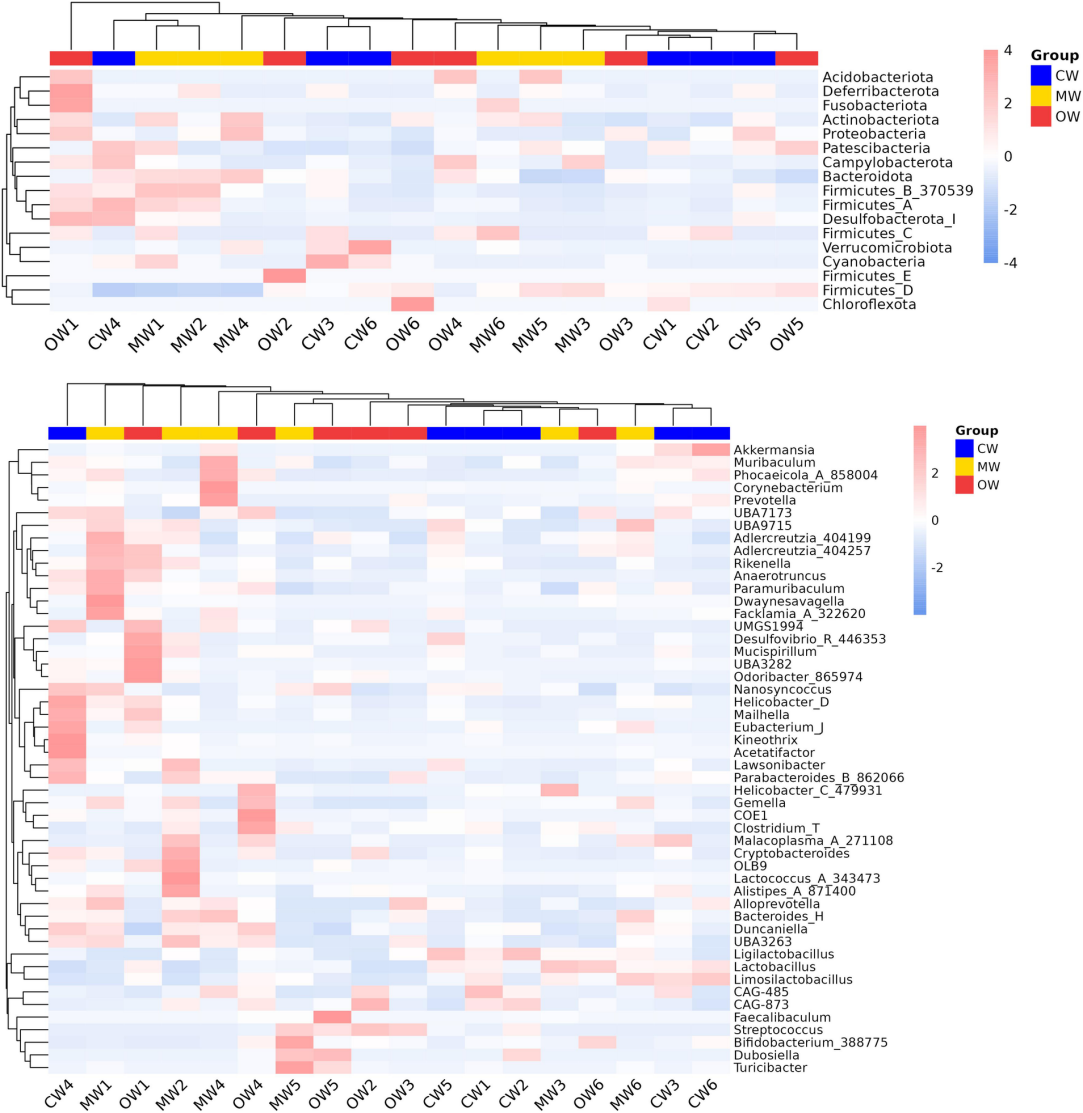
Heatmap analysis of the species abundance distribution across different levels. **(a)** Phylum and **(b)** genus.

Analysis of *β* diversity revealed non-significant differences (*p* > 0.05) among the three animal groups (Figure 4). It was found that at the phylum level, the Actinobacteriota significantly increased (*p* < 0.05) in the MW group compared with the CW group while there was a decreasing trend in the OW group. Furthermore, *Bacteroidota and Firmicutes_B_370539* exhibited similar trends in the MW and OW groups, although there were non-significant differences (*p* < 0.05) when compared with that of the control group. Conversely, *Firmicutes-D* and *Desulfobacteriota-I* showed a decreasing trend in the MW group and an increase in the OW group. Similarly, *Pelethenecus, Odoribacter,* and *Mailhella,* exhibit a reduction in abundance at the genus level within the MW group, followed by an elevation in percentages within the OW group. Furthermore, the percentages of *Faecalibaculum, UBA3789,* and *Jeotgalicoccus_A_310962* in the MW group were significantly (*p* < 0.05) higher than those in the CW group. In contrast, the percentages in the OW group were significantly higher (*p* < 0.05) than in either of the other groups. Interestingly, the levels of *Ruminococcus_E* (*p* < 0.05), *Cryptobacteroides, Muribaculum, Corynebacterium, Lactococcus, Rikenella, Alloprevotella, Bacteroides_H, Prevotella, Phocaeicola, Alistipes, and Turicibacter* increased in ALI mice, while their abundance decreased in GC-treated mice (Figures 5a,b). The above results are consistent with the heatmap results (Figure 6).

### 3.5 Metabolic pathways in APAP-induced mice

**Figure 7:**
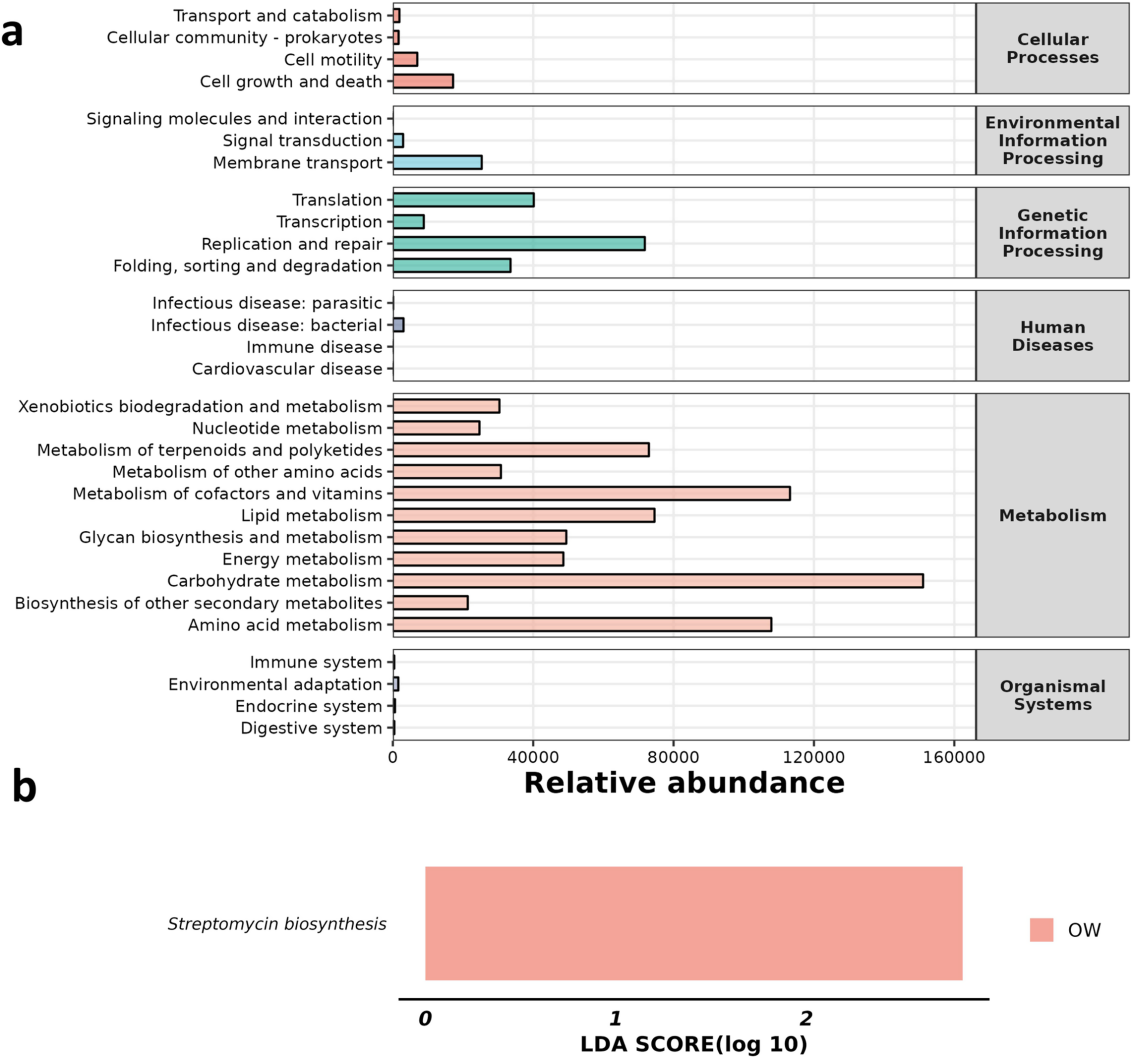
Comparison of significantly altered metabolic pathways among the three groups. **(a)** Abundance of KEGG metabolic pathway, **(b)** LDA score.

We analyzed the changes in metabolic pathways among the three groups. By comparing LDA scores, it was found that Streptomycin biosynthesis showed an upward trend in the OW group (Figure 7).

## 4 Discussion

The findings of the current study reveal that GC therapy showed a remission effect on APAP-induced ALI and resulted in increased liver indices and liver weight. This is in line with the phenomenon that ALT and AST serve as important serum transaminases and hence are indicators of hepatocyte injury (Liu et al., 2024). The elevated levels of these markers in the ALI group and the reduction in the GC group suggest that GC therapy may mitigate acute liver injury induced by APAP. IL-6, IL-10, IL-1β, and TNF-*α* are pleiotropic cytokines and pro-inflammatory factors associated with the pathogenesis of inflammation, and their alterations play a pivotal role in various acute and chronic inflammatory disorders (Xie et al., 2024). During ALI disease, there is a significant increase in the body’s inflammatory response, which can be reduced by GC treatment. In case of oxidative stress, the imbalance of oxidation and antioxidant imbalance caused by various external or internal stimuli (Sies, 2015), plays a pivotal role in toxicities. Previous *In vivo* studies reported that free radicals act on lipids to produce peroxidation, and the end product of oxidation is MDA (Tsikas, 2017). The fact explains the same phenomenon as found in our study, as “MDA content was increased significantly in APAP induction group and decreased after GC treatment.” SOD and GSH-px are the components of antioxidant enzymes in the body, and TAOC can reflect the total antioxidant capacity (Brigelius-Flohé and Flohé, 2020; Pellegrini et al., 2020; Zweier et al., 2021). The changes in the indices related to oxidative stress between the APAP induction group and the GC treatment group suggest that GC treatment is capable of mitigating oxidative stress within the body, which was consistent with a previous study (Li et al., 2023). This is an obvious fact that following acute liver injury, hepatic cell function becomes impaired, leading to oxidative stress. Moreover, prolonged or intense oxidative stress can enhance the risk of hepatocyte death (Sitbon et al., 2024). Hence, in such a scenario, the GC’s potential of alleviating oxidative stress, resulting in countering toxicity by APAP, is an appreciable novel candidate.

The two-way interaction between the gut and liver greatly depends upon the activity of gut microbiota and their metabolites, leading to eliciting a response from the liver via the portal vein, leading to the secretion of bile as the outcome (Singh et al., 2023). In the current study, APAP reduced the diversity of intestinal flora in mice, which was reduced through GC treatment, and hence, the liver restored its physiology upon application of GC. A similar type of result was presented by Dong et al. (2019), where a traditional Chinese medicine rich in dihydromyricetin (DHM) exhibited a significant hepatoprotective effect against acetaminophen (APAP)-induced liver injury (Dong et al., 2019).

The bacterial disease capacity varies greatly based on species and host as major factors, and accordingly, they might be impacting intestinal flora in one way or the other. The disturbances in the intestinal pathogens result in variations in the secretion of vital organs. *Corynebacterium,* for example, is considered among common pathogenic bacteria that have been linked to infectious diseases of kidney and lung disorders (Costa Silva et al., 2024; Li et al., 2024; Maleki-Ravasan et al., 2024), *Rikenella* on the other hand, has been responsible for body’s inflammatory response (Dai et al., 2024), and *Phocaeicola*, an anaerobic bacterium isolated from samples of brain abscesses, has been linked to nontuberculous mycobacterial lung disease (Huang et al., 2024). *Alloprevotella* has been identified as a potential biomarker for lead-induced intestinal inflammation (Wang N. et al., 2024; Wang Y. et al., 2024; Li et al., 2024). The increased abundance of these pathogens in the MW group indicates that APAP-induced acute liver injury may disturb intestinal flora and can help increase harmful flora that further affects body health. *Faecalibaculum* was found to be decreased in mice with colorectal cancer (Song et al., 2024). *Odoribacter*, a member of the Bacteroides group of anaerobic bacteria, produces short-chain fatty acids (SCFA), especially butyrate and propionate, leading to modulation of the immune system through the promotion of T-cell differentiation and inhibition of histone deacetylase, reducing pro-inflammatory cytokine expression. This can also enhance gut barrier integrity via tight junction protein expression. All these factors alleviate intestinal inflammation. SCFA also reduces apoptosis in colorectal cancer (CRC) and inhibits cancer cell proliferation, promotes differentiation of colon epithelial cells, and suppression of Wnt signaling (Xing et al., 2021). GC showed the capacity to alleviate ALI by increasing the prevalence of beneficial bacteria in the gut, and hence, the restoration of intestinal flora led to an increase in intestinal probiotics that proved to be the major factor in controlling pathogenic bacteria.

At the same time, through the analysis of metabolic pathway differences, we found that the expression of streptomycin biosynthesis was significantly increased in the OW group. The production of streptomycin, an aminocyclo-aminoglycoside antibiotic derived from *Streptomyces griseae*, marked the second instance of clinical antibiotic development following penicillin (Ishigaki et al., 2017). It functions by binding to prokaryotic ribosomes and hindering bacterial protein synthesis, thereby contributing to the inhibition or eradication of bacteria (Jiang et al., 2024; Xie et al., 2024). The observed increase in streptomycin biosynthesis within the GC treatment group suggests that GC may eliminate the inflammation caused by ALI and restore the body to normal by increasing the biosynthesis pathway of streptomycin. The synthesis of streptavidin involves the partial synthesis of inositol, which is then oxidized at C-1 and transaminated to produce trimethylinositolamine. Following phosphorylation, the compound undergoes transamination by arginine, which is then repeated at C-3. A portion of the chain sugar is synthesized from D-glucose via the DTDP-glucose pathway. Although biosynthetic gene clusters have been identified (Cuervo et al., 2023), the precise biosynthetic pathway for N-methyl-L-glucosamine fragments remains unclear. Therefore, the mechanism of how GC increases streptomycin biosynthesis remains to be studied.

## 5 Conclusion

The effects of *Galla chinensis* (GC) on acetaminophen (APAP) drug-induced acute liver injury was found promising in terms of body weight, serum profile, intestinal flora, and metabolic pathways in this study. GC brought reversal of increased liver indices caused by acute liver injury, restoration of liver functions, alleviation of oxidative stress, and reduction in inflammatory responses. Furthermore, GC partially restored disrupted structures of intestinal flora, helped in the production of beneficial flora, and enhanced streptomycin biosynthesis. The study therefore suggested GC as a suitable candidate for the optimum health of the liver, gut, and other associated parameters, including but not limited to oxidative stress and inflammation, and hence demands further investigation to formulate pharmaceutical preparations to treat diseases of the liver and gut.

## Data Availability

The original contributions presented in the study are publicly available. This data can be found at: https://www.ncbi.nlm.nih.gov/, accession number: PRJNA1131599.
